# Rhinovirus‐associated pulmonary exacerbations show a lack of FEV_1_ improvement in children with cystic fibrosis

**DOI:** 10.1111/irv.12353

**Published:** 2016-01-29

**Authors:** Mathias Cousin, Nicolas Molinari, Vincent Foulongne, Davide Caimmi, Isabelle Vachier, Michel Abely, Raphael Chiron

**Affiliations:** ^1^ Centre de Ressources et de Compétences pour la MucoviscidoseHôpital Arnaud de VilleneuveCentre Hospitalier Régional Universitaire de MontpellierMontpellierFrance; ^2^ Centre Hospitalier Régional Universitaire de MontpellierUniversité de MontpellierMontpellierFrance; ^3^ Département de StatistiquesU1046 INSERMUMR9214 CNRSCentre Hospitalier Régional Universitaire de MontpellierMontpellierFrance; ^4^ Laboratoire de virologieCentre Hospitalier Régional Universitaire de MontpellierMontpellierFrance; ^5^ INSERMU1058Centre Hospitalier Régional Universitaire de MontpellierMontpellierFrance; ^6^ Centre de Ressources et de Compétences pour la MucoviscidoseAmerican Memorial HospitalReims CedexFrance

**Keywords:** children, cystic fibrosis, pulmonary exacerbations, respiratory viruses, rhinovirus

## Abstract

**Background:**

Respiratory viral infections lead to bronchial inflammation in patients with cystic fibrosis, especially during pulmonary exacerbations. The aim of this study was to determine the impact of viral‐associated pulmonary exacerbations in children with cystic fibrosis and failure to improve forced expiratory volume in 1 s (FEV_1_) after an appropriate treatment.

**Methods:**

We lead a pilot study from January 2009 until March 2013. Children with a diagnosis of cystic fibrosis were longitudinally evaluated three times: at baseline (Visit 1), at the diagnosis of pulmonary exacerbation (Visit 2), and after exacerbation treatment (Visit 3). Nasal and bronchial samples were analyzed at each visit with multiplex viral respiratory PCR panel (qualitative detection of 16 viruses). Pulmonary function tests were recorded at each visit, in order to highlight a possible failure to improve them after treatment. Lack of improvement was defined by an increase in FEV_1_ less than 5% between Visit 2 and Visit 3.

**Results:**

Eighteen children were analyzed in the study. 10 patients failed to improve by more than 5% their FEV_1_ between Visit 2 and Visit 3. Rhinovirus infection at Visit 2 or Visit 3 was the only risk factor significantly associated with such a failure (OR, 12; 95% CI, 1·3–111·3), *P* = 0·03.

**Conclusions:**

Rhinovirus infection seems to play a role in the FEV_1_ recovery after pulmonary exacerbation treatment in children with cystic fibrosis. Such an association needs to be confirmed by a large‐scale study because this finding may have important implications for pulmonary exacerbation management.

## Introduction

Despite conventional management with antibiotics and respiratory physiotherapy, pulmonary function undergoes a steady decline in cystic fibrosis (CF). Pulmonary exacerbations (PEx) are an important clinical outcome in CF, as they are associated with a faster rate of decline in forced expiratory volume in 1 s (FEV_1_) and with an increased morbidity and mortality in patients with CF. Almost 25% of patients fail to recover to baseline FEV_1_ after a PEx.^1^, ^2^ The reasons why these patients fail to recover previous FEV_1_ after an appropriate treatment are not fully understood even though some risk factors have been identified (pancreatic insufficiency, low body mass index (BMI), chronic infection with CF pathogens, greater drop in FEV_1_ from baseline at treatment initiation).^3^ Viruses are frequent triggers of PEx, and rhinoviruses are the most frequent viral agents found in patients with CF.^4^ An impaired innate host defense may cause an increased susceptibility to viral respiratory infection in patients with CF, and some severe viral infections might be responsible for the exaggerated pulmonary inflammatory response.^5^, ^6^

However, in the literature, there is a paucity of data regarding PEx outcomes in CF children, with a concomitant viral respiratory infection. These infections seem to play a significant role in pulmonary morbidity and inflammation in patients with CF. Indeed, patients presenting with concomitant bacterial and viral infections show a greater rate of both hospitalization and antibiotic prescription.^7^ Unfortunately, the consequences of viral respiratory infections on the evolution of CF lung disease are hard to study on a large scale, because, in order to evaluate such an issue, a close longitudinal follow‐up with repeated viral swab assessment is needed. We could therefore speculate that the impact of respiratory viruses is probably underestimated in patients with CF.

We hypothesized that respiratory viruses are major risk factors for maintaining and extending bronchial inflammation during the PEx recovery period. The aim of this study was to determine the association between viral‐associated PEx and failure to improve FEV_1_ after PEx treatment in CF children.

## Materials and methods

### Study design

This pediatric pilot study was conducted from January 2009 until March 2013 at the CF centers of Montpellier and Reims (France). Patients aged from 7 to 18 years were recruited. PEx was defined, according to European Respiratory Society criteria, as a change in respiratory status requiring antibiotic treatment, or by a cluster of symptoms, as indicated by EuroCareCF Working Group. The study EudraCT (2008‐00451‐30) has been approved by our local ethic committee (ref 2008.07.06bis). All children and both parents signed the consent form. Patients were longitudinally assessed three times in the CF university center: at baseline (Visit 1, V1), at the diagnosis of PEx (Visit 2, V2), and after PEx treatment (Visit 3, V3). V3 was scheduled between 2 and 6 weeks after the beginning of PEx treatment, because it has been shown that antibiotic response to exacerbation as assessed by PFT is essentially complete 2 weeks after treatment initiation.^9^ Clinicians working in CF university centers managed PEx, according to the guidelines.

### Collection of clinical information and specimens

Clinical data and PFT were recorded at each visit. We sampled upper and lower airways by collecting two nasal swabs and two spontaneously expectorated sputa at each visit for viral and bacterial analysis. Viral analysis was performed through a commercial multiplex PCR assay (Anyplex‐II‐RV16, Seegene) providing a qualitative detection of 16 viruses (without genotype): human adenovirus; influenza A and B virus; human parainfluenza virus 1, 2, 3, and 4; human rhinovirus; human respiratory syncytial virus A and B; human bocavirus; human coronavirus 229E, NL63, and OC43; human metapneumovirus; and human enterovirus. Clinicians were blinded to the results of virological tests until the end of the study.

### Statistical analysis

Participants were categorized by their relative change in FEV_1%_ predicted between Visit 2 and Visit 3 calculated as follows:([FEV_1%_ predicted at Visit 2‐FEV_1%_ predicted at Visit 3] * 100)/FEV_1%_ predicted at Visit 2; as “responders” (≥5% of FEV_1_ improvement) and “non‐responders” (<5% of FEV_1_ improvement) to PEx treatment. This 5% threshold has been previously used in many studies.^10^, ^11^

Quantitative variables were expressed as means (standard deviation) and compared using the Wilcoxon test as appropriate. Qualitative variables were expressed as numbers (%), and the absolute numbers were compared using the chi‐squared test or the Fisher's test as appropriate. Logistic regressions were used to estimate OR and 95% CI.

## Results

### Study population

In the present study, 34 patients were included (21 in Montpellier center and 13 in Reims center). Among these patients, 18 completed the 3 visits and were analyzed. The other patients were lost to follow up because they did not go on exacerbation during the period of the study. Eight patients were classified as responders and 10 patients as non‐responders to PEx treatment. Patient characteristics were similar between the two groups (Table 1). Patients did not present severe associated comorbidities: none had diabetes, one patient was undernourished, and one patient had a FEV_1_ below 60% at baseline. The delay between V2 and V3 varied from 13 to 50 days (median delay of 29 days), without any difference between the two groups (Table 1). In the non‐responders’ group, there were significantly more viral respiratory infections at V2 and/or V3 (10 versus 4, *P* = 0·0229) and more rhinovirus respiratory infections at V2 and/or V3 (8 versus 2, *P* = 0·0196) (Table 1). At Visit 2, we detected viruses in 50% of nasal swabs and in 67% of expectorated sputa. Nasal and bronchial swabs were concordant in 78% of all visits.

**Table 1:** Baseline demographic data in responders and non‐responders

Characteristics	Non‐responders (*n *= 10)	Responders (*n *= 8)	All patients (*n *= 18)	*P*‐value
Age at inclusion (in years)	11·90 ± 3·17	13·12 ± 2·58	12·44 ± 2·91	0·5012
Males (%)	8 (80·00)	5 (62·50)	13 (72,22)	0·61
Homozygous F508del (%)	5 (50·00)	4 (50·00)	9 (50·00)	1
Pancreatic insufficiency (%)	9 (90·00)	7 (87·50)	16 (88·89)	1
BMI percentile	53·50 ± 35·43	26·12 ± 20·22	41·33 ± 32·08	0·1424
Atopic status	1 (10,00)	2 (25,00)	3 (16,67)	0·5598
Delay between V2 and V3 (in days)	31·60 ± 11·02	26·25 ± 8·33	29·22 ± 10·02	0·3710
Chronic PA infection (%)	4 (40·00)	4 (50,00)	8 (44·44)	1
Chronic SAMS infection (%)	9 (90·00)	5 (62·50)	14 (77·78)	0·27
V1 FEV_1%_ predicted	91·57 ± 20·42	90·99 ± 15·02	91·31 ± 17·71	0·6893
V2 FEV_1%_ predicted	81·05 ± 19·39	74·63 ± 22·14	82·42 ± 21·18	0·1200
V3 FEV_1%_ predicted	73·03 ± 15·12	85·86 ± 16·33	83·29 ± 16·70	0·1426
Presence of respiratory virus in airways at V2 and/or V3 (%)	10 (100·00)	4 (50·00)	14 (77·78)	0·0229
Presence of rhinovirus in airways at V2 and/or V3 (%)	8 (80·00)	2 (25·00)	10 (55·56)	0·0196

FEV_1_, forced expiratory volume in 1 s; V1, Visit 1; V2, Visit 2; V3, Visit 3; SAMS, *Staphylococcus aureus* methicillin‐sensitive; PA, *Pseudomonas aeruginosa*.

Responders and non‐responders were compared using a chi‐squared or exact Fisher's test.

The proportion of viral respiratory infection was 72% at the diagnosis of PEx (V2), 22% at baseline (V1), and 28% at V3. Rhinoviruses accounted for 64% of identified viruses. We detected a viral co‐infection in one patient (rhinovirus and metapneumovirus). Influenza virus vaccination was recorded for 78% of the patients. PEx did not occur during any specific respiratory viruses epidemic spread or a specific season**.**

### Exploratory data analysis

The only risk factor significantly associated with failure to improve FEV_1_ above 5% after PEx was viral respiratory infection at V2 and/or V3 (OR, 2·04; 95% CI, 1·57–2·67) *P* = 0·0088 and especially with rhinovirus infection: (OR, 12; 95% CI, 1·29–111·32, *P* = 0·0288).

Other factors such as pancreatic insufficiency, atopic status, BMI, baseline treatment (azithromycin, inhaled corticosteroid therapy, and antibiotic therapy), persistent infection (with *Pseudomonas aeruginosa*,* Staphylococcus aureus),* detection of new bacteria at V2 (all pathogens included and for each bacteria species), spirometrics parameters (i.e., FEV_1%_ drop between V1 and V2) and therapeutic features (i.e., adapted PEx antibiotherapy according to ECFS guidelines 2014,^12^ intravenous treatment, duration of antibiotic treatment) were not significantly associated with failure to improve FEV_1_ above 5% after PEx treatment (Table 2).

**Table 2:** Univariate model for the association between failure to improve FEV_1_ ≥ 5% between V2 and V3 and other variables

Variables	Odds ratio	95% CI		*P*‐value
Number of days between V2 and V3	1·06	0·95	1·19	0·2653
New bacteria at V2	9·00	0·75	108·31	0·0834
Adapted antibiotic therapy at V2	0·57	0·04	7·74	0·6739
Number of antibiotics used to treat exacerbation	0·26	0·06	1·18	0·0804
Intravenous antibiotic administration	0·19	0·02	2·29	0·1885
Duration of exacerbation treatment (days)	0·94	0·83	1·07	0·3630
Viral respiratory infection at V2	9·00	0·75	108·31	0·0834
Viral respiratory infection at V3	1·29	0·16	10·45	0·8141
Rhinovirus respiratory infection at V2	4·50	0·59	34·61	0·1484
Rhinovirus respiratory infection at V3	1·75	0·13	23·70	0·6739
Viral respiratory infection at V2 and/or V3	**2·04**	1·57	2·67	**0·0088**
Rhinovirus respiratory infection at V2 and/or V3	**12·00**	1·29	111·32	**0·0288**
Increase in FVC > 5% between V2 and V3	**0·07**	0·01	0·82	**0·0347**

FEV_1_, forced expiratory volume in 1 s; FVC, forced vital capacity; V1, Visit 1; V2, Visit 2; V3, Visit 3.

Bold values indicate significant results.

### Other PFT parameters

Non‐responder patients failed significantly to improve above 5% their forced vital capacity (FVC) between V2 and V3, (OR, 0·07; 95% CI, 0·01–0·82), *P* = 0·0347 (Table 2). Rhinovirus respiratory infection at V2 or V3 was significantly associated with failure to improve FVC above 5% after PEx treatment (adjusted OR, 15; 95% CI, 1·21–185·2), *P* = 0·0347.

## Discussion

The results of this pilot study show that rhinovirus infection during PEx is significantly associated with failure to improve FEV_1_ after PEx treatment in children with CF.

None of our subjects had new symptoms of a pulmonary exacerbation at the third visit. So we do not think that the non‐responder's children were sick again but this failure in FEV1 recovery might be the consequences of the exacerbation diagnosed at the second visit.

To our knowledge, only one study assessed the relationship between respiratory viruses (sampled by bronchoscopy) and PFT results after PEx treatment in CF pediatric patients, and they observed a strong association between respiratory viruses and newly acquired common respiratory pathogens and a worse recovery of FEV1 in the months following bronchoscopy for patients infected with respiratory viruses.^13^ We performed a complete non‐invasive assessment of viral respiratory infection in both upper and lower airways; such a method was useful because in 9% of the collected specimens, viral analysis was negative by nasal swab, but positive in sputum.

Virus and mainly rhinoviruses are associated with asthma exacerbations. In our study, one patient had asthma and was was treated with inhaled corticotherapy.

The main limitation of our study is that we included a small number of patients and that we could have designed a fourth visit later to follow the FEV1 of non‐responder's children. Moreover, our microbiological analysis was not exhaustive, because atypical mycobacteria, anaerobic bacteria, and fungi were not investigated.

Some studies show that, in the upper airways, rhinovirus‐associated clinical symptoms are more likely the result of local and systemic immune responses than a consequence of direct cytopathogenic effects.^14^ Nevertheless, the pathophysiology of rhinovirus‐associated PEx remains unclear in CF, and two recent studies showed an exaggerated bronchial inflammation associated with rhinovirus infection in airways epithelial cells in patients with CF.^15^

Further large‐scale analyses are required to study the impact of rhinoviruses on failure to recover to previous FEV_1_ levels in CF children.

## Conflicts of interest

None.
